# Tolerability of a Fully Maturated Cheese in Cow’s Milk Allergic Children: Biochemical, Immunochemical, and Clinical Aspects

**DOI:** 10.1371/journal.pone.0040945

**Published:** 2012-07-19

**Authors:** Claudia Alessandri, Stefano Sforza, Paola Palazzo, Francesca Lambertini, Sara Paolella, Danila Zennaro, Chiara Rafaiani, Rosetta Ferrara, Maria Livia Bernardi, Mario Santoro, Sara Zuzzi, Ivana Giangrieco, Arnaldo Dossena, Adriano Mari

**Affiliations:** 1 Center for Molecular Allergology, IDI-IRCCS, Rome, Italy; 2 Department of Organic and Industrial Chemistry, University of Parma, Parma, Italy; Leiden University Medical Center, The Netherlands

## Abstract

**Background:**

From patients’ reports and our preliminary observations, a fully maturated cheese (*Parmigiano-Reggiano;* PR) seems to be well tolerated by a subset of cow’s milk (CM) allergic patients.

**Objective and Methods:**

To biochemically and immunologically characterize PR samples at different maturation stage and to verify PR tolerability in CM allergic children. Seventy patients, with suspected CM allergy, were enrolled. IgE to CM, α-lactalbumin (ALA), β-lactoglobulin (BLG) and caseins (CAS) were tested using ImmunoCAP, ISAC103 and skin prick test. Patients underwent a double-blind, placebo-controlled food challenge with CM, and an open food challenge with 36 months-maturated PR. Extracts obtained from PR samples were biochemically analyzed in order to determine protein and peptide contents. Pepsin and trypsin-chymotrypsin-pepsin simulated digestions were applied to PR extracts. Each PR extract was investigated by IgE Single Point Highest Inhibition Achievable assay (SPHIAa). The efficiency analysis was carried out using CM and PR oral challenges as gold standards.

**Results:**

The IgE binding to milk allergens was 100% inhibited by almost all PR preparations; the only difference was for CAS, mainly α_S1_-CAS. Sixteen patients sensitized to CM tolerated both CM and PR; 29 patients tolerated PR only; 21 patients, reacted to both CM and PR, whereas 4 patients reactive to CM refused to ingest PR. ROC analysis showed that the absence of IgE to BLG measured by ISAC could be a good marker of PR tolerance. The SPHIAa using digested PR preparations showed a marked effect on IgE binding to CAS and almost none on ALA and BLG.

**Conclusions:**

58% of patients clinically reactive to CM tolerated fully maturated PR. The preliminary digestion of CAS induced by PR maturation process, facilitating a further loss of allergenic reactivity during gut digestion, might explain the tolerance. This hypothesis seems to work when no IgE sensitization to ISAC BLG is detected.

## Introduction

Cow’s milk allergy (CM) is the most common food allergy during early infancy. CM contains allergenic proteins, caseins (CAS) and whey proteins, being the former a fraction accounting for 80% of total CM proteins, and including four different proteins: α_S1_-, α_S2_-, β- and κ-CAS [1]–[3]. β-lactoglobulin (BLG), whose homolog is not present in human milk, represents 50% of the CM whey proteins. The first line treatment for CM allergy is CM and dairy products avoidance [4], [5], sometime leading to growth impairment [4]. Hydrolyzed formulas are good alternatives in CM allergic children though not always well accepted because of their not excellent taste [6]. They are produced by combined enzymatic and heat treatments and ultra-filtration of cow’s milk proteins. According to the degree of protein modifications, hydrolyzed CM formulas are differentiated in partially and extensively hydrolyzed whey or CAS hydrolysates. Studies have supported the safety of including extensively heated products containing milk into the diet for those patients who are non-reactive [7].

Beside the clinical history and the oral food challenge, the diagnostic workup to correctly identify CM protein allergic patients is based on the use of *in vivo* or *in vitro* approaches, skin test and IgE detection respectively. Both approaches can be based on a fresh milk preparation (prick-prick test) and CM extracts [5], or discrete CM allergenic molecules [3]. The *in vitro* testing using allergenic molecules can be performed either in a singleplex way, like most of the currently used laboratory systems (*e.g.* ImmunoCAP, Immulite) or by multiplex testing using the allergenic molecule-based microarray technology (*e.g.* ISAC system) [8], [9]. The latter laboratory approach allows IgE detection for a panel of CM-related and non-related allergenic molecules in a single run giving a broader view on patients’ sensitization profiles [8], [10].

*Parmigiano-Reggiano* (PR) is an Italian CM-based cheese with a variable but long and natural maturation process (12–36 months), produced in the place of origin and appointed for that with the “Protected Designation of Origin - PDO”. Production is carried out under rigid specifications (http://www.parmigianoreggiano.com/consortium/rules_regulation_2/specification_single_document.aspx). The milk used in the process, obtained by cows fed with locally grown forage, is monitored to ensure the high quality and the presence of special characteristics. One kg of PR is made using 16 litres of CM. The partially skimmed milk is poured into copper cauldrons where calf rennet and fermented whey, rich in natural lactic ferments obtained from the processing of the day before, are added. The milk coagulates and the curd is then broken down into minuscule granules. After a cooking process, at 55°C, the cheesy granules form a single mass that will be placed in a mould which will give PR its final shape. After a few days the process of salting closes the production cycle and opens the maturation cycle. During maturation rennet’s and lactic acid bacteria’s proteases digest milk proteins. The full production cycle releases a cheese having a content of 30% water and 70% nutrients [11], mainly constituted by highly hydrolyzed proteins [12]. Different production procedures and maturation length adopted by other Italian and non-Italian parmesan producers give raise to different biochemical products as showed by Sforza et al. [12].

During the maturation, CAS are gradually and constantly broken down by the proteolytic enzymes of milk, rennet and lactic acid bacteria, yielding a nitrogen fraction which is continuously changing during the cheese maturation, gradually shifting from full proteins to longer peptides, then to shorter peptides and free amino acids. The peptide fraction of aged cheese is thus extremely complex, being formed by proteins, hundreds of different peptides, mostly deriving from α_S1_- and β-CAS, the two most abundant proteins in the CAS fraction, and free amino acids. The composition of this fraction is related to the month of maturation, and also, to a lesser extent, to the single producer [12], [13].

Up to now allergenicity of PR proteins has never been studied. From patients’ and caregivers’ reports and from our preliminary observations, original PR cheese seemed to be well tolerated by a subset of CM allergic patients unless having generalized reactions on CM protein exposure.

In the present study, we sought to define the clinical tolerability of PR in CM allergic patients, characterizing the biochemical, immunochemical, and clinical aspects of the condition, trying to understand the underlying mechanisms and searching for a possible marker of PR reactivity/tolerability.

## Methods

### Patients’ Selection and Allergy Testing

The study was carried out at the Center of Molecular Allergology, IDI-IRCCS, Rome, Italy, during the period December 2009–June 2011. Seventy-five patients referred to our center by the family pediatricians for suspected CM allergy have been enrolled. Seventy patients (47 male), age ranging from 6 months to 16 years (3 years old median age), agreed to participate. The suspicious of CM allergy was based on history of reactions after ingestion or contact with CM, or positive SPT or IgE to CM extracts. At the time of consultation, all patients were following a CM elimination diet set before by other physicians. All *in vivo* and *in vitro* tests performed in the present study are part of the routine diagnostic workup for CM allergic patients. Institutional Ethics Committee approval was obtained for using data, biological material, and performs PR challenge (29/CE/2009). Informed signed consent to perform food challenges was obtained from caregivers. *In vivo* tests have been performed in a highly protected environment in the day care centre.

Demographics, clinical data, namely respiratory and ingestion-related symptoms on CM exposure, and *in vivo* and *in vitro* test results, were recorded for all patients by an allergy specialist using the InterAll software, a customized allergy electronic record for diagnostic and clinical data storing (version 3.0, Allergy Data Laboratories, Latina, Italy).

Specifications on allergenic proteins used in the present study are given in Table 1. SPT were performed and measured as previously reported [10] using fresh skimmed CM, α-lactalbumin (ALA), BLG, CAS, and milk extract commercial preparations (Lofarma, Milan, Italy). A wheal area greater than 7 mm^2^ was scored positive. Specific IgE towards CM, ALA, BLG and CAS were measured using the ImmunoCAP system (Phadia, Sweden); positive values were deemed when >0.35 kUa/l. Specific IgE for the same three allergen preparations *plus* bovine serum albumin (BSA) were measured using the ISAC 103 (Phadia Multiplexing Diagnostics, PMD, Vienna, Austria) as previously reported [14]; positive threshold value was set at 0.01 kUa/l.

**Table 1:** Cow’s milk allergenic protein nomenclature and short names as adopted within the present study.

Milk Allergen Nomenclature					Used for Diagnosis and Experiments#			
Common Name	ShortName°	WHO-IUIS Nomenclature*	Allergome Name§	Allergome Code§	Skin PrickTest	CAP	ISAC 103	ISAC 89
Cow’s Milk	CM	N.A.	Bos d [Milk]	1747	Tested	Tested	N.T.	N.T.
α-Lactalbumin	ALA	Bos d 4	Bos d 4	163	Tested	Tested	Tested	Tested
β-lactoglobulin	BLG	Bos d 5	Bos d 5	164	Tested	Tested	Tested	N.T.
β-lactoglobulin A	BLG-A	N.A.	Bos d 5.0102	2738	N.T.	N.T.	N.T.	Tested
β-lactoglobulin B	BLG-B	Bos d 5.0101	Bos d 5.0101	2739	N.T.	N.T.	N.T.	Tested
Serum Albumin	BSA	Bos d 6	Bos d 6	165	N.T.	N.T.	Tested	Tested
Lactoferrin	LCF	N.A.	Bos d Lactoferrin	1065	N.T.	N.T.	Tested	Tested
Caseins	CAS	Bos d 8	Bos d 8	167	Tested	Tested	Tested	Tested
α_s1_-Casein	α_s1_-CAS	N.A.	Bos d 8 alphaS1	2734	N.T.	N.T.	N.T.	Tested
α_s2_-Casein		N.A.	Bos d 8 alphaS2	2735	N.T.	N.T.	N.T.	N.T.
β-Casein	β-CAS	N.A.	Bos d 8 beta	2736	N.T.	N.T.	N.T.	Tested
κ-Casein	κ-CAS	N.A.	Bos d 8 kappa	2737	N.T.	N.T.	N.T.	Tested

Allergen specifications for each diagnostic or experimental testing are included.

°As used in the present paper;

* www.allergen.org.

§ www.allergome.org.

# The test for diagnosis and experiments were provided as follows: Skin prick test by Lofarma, excepting cow’s milk which was also used as fresh skimmed preparation by prick-prick testing; CAP by Phadia; ISAC 103 by PMD; ISAC 89 by VBC-Genomics;

N.A.: Not available, N.T.: not tested in the present study.

### Food Challenges and Tolerance Follow Up

In order to verify the consistency of CM allergy diagnosis, a double blind placebo controlled CM challenge (DBPCFC) has been carried out in patients without recent CM anaphylaxis using fresh pasteurized skimmed milk (3.5% fat, 3.2 gr proteins/100 ml ) following current international recommendations [15], [16]. A preparation of 150 ml of skimmed milk and two spoons of white sugar were used as *verum*. The same preparation without CM but with soy or rice milk, depending on patient’s use, was served as placebo. To mask smell, taste, and consistency differences, three teaspoons of sugarless cocoa (Cacao Amaro, Perugina, Italy), free of any nut contamination, were used for all preparations. In order to verify PR tolerability within the subset of CM allergic children, patients underwent challenge with 36 months matured PR preparation provided as a single lot from a single dairy by the PR consortium, and kept frozen at −20°C until used. An open food challenge procedure (OFC) has been adopted as it was impossible to adequately mask by any mean the strong PR taste to perform a DBPCFC. Patients were admitted to our centre in the morning, in a fasting state; administration of antihistamines, if any, was stopped at least one week prior to skin test and oral challenge, in order to avoid any effect on test outcomes. None of the patients was under steroid treatment. Blinded active and placebo meals were randomly administered in identical doses on separate days, prepared immediately before challenge [15], [16]. CM challenge was performed as shown in Table 2, administering increasing doses every 20 minutes. PR challenge was performed taking into account the estimated CM proteins equivalent of 200 ml CM  = 13.3 gr PR, as shown in Table 2. Only symptoms appearing soon after the challenge have been considered, whereas eczema flare up appearing the following days to the oral challenge was not considered as any objective evaluation was possible.

**Table 2:** Oral Challenge schedules in patients with reported cow’s milk sensitization.

	Cow’s Milk*		*Parmigiano-Reggiano*°		
Dose	ml	Amount	Gr	Amount	
1	0.05	1 drop	0.003	1 drop	PR (1 gr) *plus* water (14 ml)
2	0.15	3 drops	0.01	3 drops	
3	0.3	6 drops	0.02	6 drops	
4	1	1 ml	0.07	1 ml	
5	3	3 ml	0.2	3 ml	
6	10	10 ml	0.7	10 ml	
7	30	30 ml	2.00	2 gr	PR as is
8	50	50 ml	3.03	3 gr	
9	100	100 ml	6.07	7 gr	
Cumulative	195		13.0		

* Cow’s Milk: Double Blind Placebo Controlled Food Challenge;

°*Parmigiano-Reggiano*: Open Food Challenge;

PR: *Parmigiano-Reggiano* cheese;

Protein equivalence (aprox): 200 ml Cow’s Milk  = 13.3 gr PR.

PR tolerant subjects were invited to continue consuming the 36 months maturated PR by using commercially available preparations as certified by the PR consortium. Symptoms recording in the follow up phase was made possible by using scheduled control visit attendance, or, if the visit was delayed for any reason, by email contacts or telephone calls.

### Preparation of Water Soluble Extracts of PR Cheese

Forty six PR samples (1.5 kg each), at maturation ages between 6 and 41 months, were received from six producers as detailed in Table 3. Ninety two water soluble extracts (WSE) were obtained from the 46 PR samples and were prepared by homogenizing 20 gr of finely grated cheese by Ultra Turrax T50 Basic (IKA-Werke, Staufen, Germany) for 1.5 minutes at 4000 rpm in 90 ml of deionised water. The mixture was then filtered through filter paper to obtain a limpid extract.

**Table 3:** Ninety two *PR* water soluble extracts from 46 samples as provided by six producers at different maturation ages.

Sample	Protein(mg/ml)	Sample	Protein(mg/ml)	Sample	Protein(mg/ml)	Sample	Protein(mg/ml)	Sample	Protein(mg/ml)
**A-6-1-a**	2,19	A-6-2-a	1,71	**C-6-1-a**	1,43	C-6-2-a	0,89	E-6-1-a	0,82
A-12-1-a	2,18	A-12-2-a	1,21	C-14-1-a	0,93	C-13-2-a	0,97	E-12-1-a	1,26
**A-16-1-a**	1,88	A-16-2-a	1,05	**C-18-1-a**	1,14	C-18-2-a	1,14	E-24-1-a	0,98
A-24-1-a	1,17	A-24-2-a	2,00	C-24-1-a	0,91	C-23-2-a	1,12	E-37-1-a	0,73
**A-36-1-a**	1,55	A-36-2-a	2,17	**C-38-1-a**	1,06			E-41-1-a	0,72
A-6-1-b	1,27	A-6-2-b	2,35	C-6-1-b	1,42	C-6-2-b	1,02	E-6-1-b	0,85
A-12-1-b	1,76	A-12-2-b	1,97	C-14-1-b	2,03	C-13-2-b	1,21	E-12-1-b	1,18
A-16-1-b	1,05	A-16-2-b	2,44	C-18-1-b	1,26	C-18-2-b	1,10	E-24-1-b	0,98
A-24-1-b	1,36	A-24-2-b	1,88	C-24-1-b	1,85	C-24-2-b	1,41	E-37-1-b	0,80
A-36-1-b	1,38	A-36-2-b	2,01	C-38-1-b	1,15			E-41-1-b	0,80
**B-6-1-a**	1,37	B-6-2-a	1,20	**D-7-1-a**	1,37	D-6-2-a	1,43		
B-12-1-a	2,60	B-12-2-a	1,29	D-12-1-a	1,37	D-12-2-a	1,20	F-11-1-a	1,43
**B-18-1-a**	2,10	B-18-2-a	1,14	**D-20-1-a**	1,67	D-24-2-a	1,21	F-24-1-a	1,66
B-25-1-a	2,00	B-24-2-a	1,25	D-26-1-a	1,07	D-36-2-a	1,37	F-33-1-a	1,75
**B-36-1-a**	2,39	B-36-2-a		**D-41-1-a**	1,12				
B-6-1-b	1,37	B-6-2-b	1,17	D-7-1-b	1,35	D-6-2-b	1,15		
B-12-1-b	1,75	B-12-2-b	1,06	D-12-1-b	1,64	D-12-2-b	0,99	F-11-1-b	2,10
B-18-1-b	1,09	B-18-2-b	1,49	D-20-1-b	1,54	D-24-2-b	1,11	F-24-1-b	1,61
B-25-1-b	1,33	B-24-2-b	1,21	D-26-1-b	1,13	D-36-2-b	1,35	F-33-1-b	1,94
B-36-1-b	1,40	B-36-2-b	1,46	D-41-1-b	1,11				

One or two samples were provided, and one or two water soluble extracts were prepared.

Footnote: Each water soluble extract has been identified using an uppercase letter for the producer, a number indicating the maturation age in months, a second number indicating the first or second sample when available, and a lowercase letter indicating the performed extractions. Protein yield in mg/ml are given for each extract. Extracts marked in bold have been used for the simulated gastrointestinal digestion experiments.

### SDS-PAGE of PR Water Soluble Extracts

SDS-PAGE was performed on CriterionTM XT Precast gels 12% Bis-Tris (Bio Rad) according to the standard procedures indicated by the manufacturer (Bio-Rad). 100 µl of WSE were dried by nitrogen flow, re-dissolved in 25 µl of reducing sample buffer, and incubated at 95°C for 5 minutes. About 1 mg of ALA, BLG, α-CAS, β-CAS and κ-CAS were dissolved in 1 ml of deionised water and the volume corresponding to 5 µg of each protein was dried under nitrogen flow. Solid residue was dissolved in 25 µl of reducing sample buffer and incubated at 95°C for 5 minutes. The run was performed at 150 V and lasted about 55 minutes, using 1% of Coomassie blue in the sample buffer as indicator. The gel was transferred into a vessel with the staining solution covering it and placed in swelling for at least 1 hour. After that, the gel was rinsed with de-staining solution in order to achieve the desired contrast. Gel was acquired by GS-800 Calibrated Densitometer with Quantity One software (Bio Rad).

### Peptide and Whey Protein Determination by UHPLC/ESI-MS Analysis

For UHPLC analysis, 900 µl of WSE were transferred into a vial and dried under nitrogen flow, then dissolved in 150 µl, of 0.1% formic acid solution. UHPLC/ESI-MS analysis was performed by using an Acquity UPLC separation system (Waters, Milford, Ma, USA) with an Acquity BEH C18 2.1×150 mm column (Waters), 1.7 µm particle size, according to a previous published methodology [Sforza S, Cavatorta V, Lambertini F, Galaverna G, Dossena A et al. J Dairy Sci 2012; accepted for publication]. Each UHPLC/ESI-MS chromatogram was analyzed in order to determine the molecular weight and the characteristic ions of the main peaks. In the case of molecular weights higher than 1 kDa, reconstructed mass peaks were obtained by MaxEnt 1, application of Masslynx 4.0 software (Waters, Milford, Ma, USA). Peptides were mostly identified according to their MW and to fragments present in the mass spectra, according to a methodology previously developed [12], [13], also using a suitably developed software for assisting the identification, by narrowing the possibilities according to the proteins generating the proteolytic peptides (Pep Sirio software, Biodigital Valley, Italy).

The quantification of ALA and BLG in the extracts was performed by UHPLC/ESI-MS applying the same method as above, with the external standard method for quantification. Calibration curves were obtained by injecting in the UHPLC/ESI-MS systems standard solutions of ALA and BLG at different concentrations. All standards and samples were analyzed by extracting from the full scan chromatograms the selected ion chromatograms for the two proteins, by using the following ions: m/z 1418.3, 1575.8 for ALA; m/z 1143.0, 1219.1 for BLG-B, and 1148.3, 1224.9 for BLG-A. The chromatographic peaks were integrated in all the extracted chromatograms, and the standard solutions were used as reference for the quantification of the same proteins in the samples.

### In vitro Simulated Gastrointestinal Digestion of PR Water Soluble Extracts

Two different digestion models were applied: the first simulating the gastric transit, using pepsin, and a second one using all three major digestive enzymes, namely trypsin and chymotripsin after pepsin treatment (TCP), in order to simulate the digestion in the gastric and the intestinal tracts. The 12 WSE marked in Table 3 were used in the digestion experiments. For preparing standard solutions, 10 mg of pepsin were dissolved in 10 ml of hydrochloric acid solution at pH 4.4 and stored at −20°C, 10 mg of trypsin were dissolved in 10 ml of hydrochloric acid solution 1 mM at pH 3.0 and stored at −20°C, 10 mg of chymotrypsin were dissolved in 10 ml of hydrochloric acid solution 1 mM containing CaCl_2_ 2 mM and stored at −20°C. 5 ml of WSE were acidified to pH 2.2 by the addition of hydrochloric acid 1 mM and 50 µl of pepsin stock solution were added (enzyme:protein ratio 1∶100). The mixture was incubated in horizontal swelling for 3 hours at 37°C in order to simulate the gastric transit. The solution was then neutralized by the addition of NH_4_HCO_3_ to pH 7.5 and 50 µl of trypsin and 50 µl of chymotrypsin stock solutions (enzyme:protein ratio 1∶100) were then added. The mixture was incubated in horizontal swelling for 4 hours at 37°C in order to simulate intestinal transit. In order to stop the digestion at the end of the simulation, 1 ml of acetonitrile was mixed with the solution. Finally, 1 ml of digested WSE was centrifuged at 16000 *g* for 15 minutes. The supernatant was stored at −20°C.

**Figure 1:**
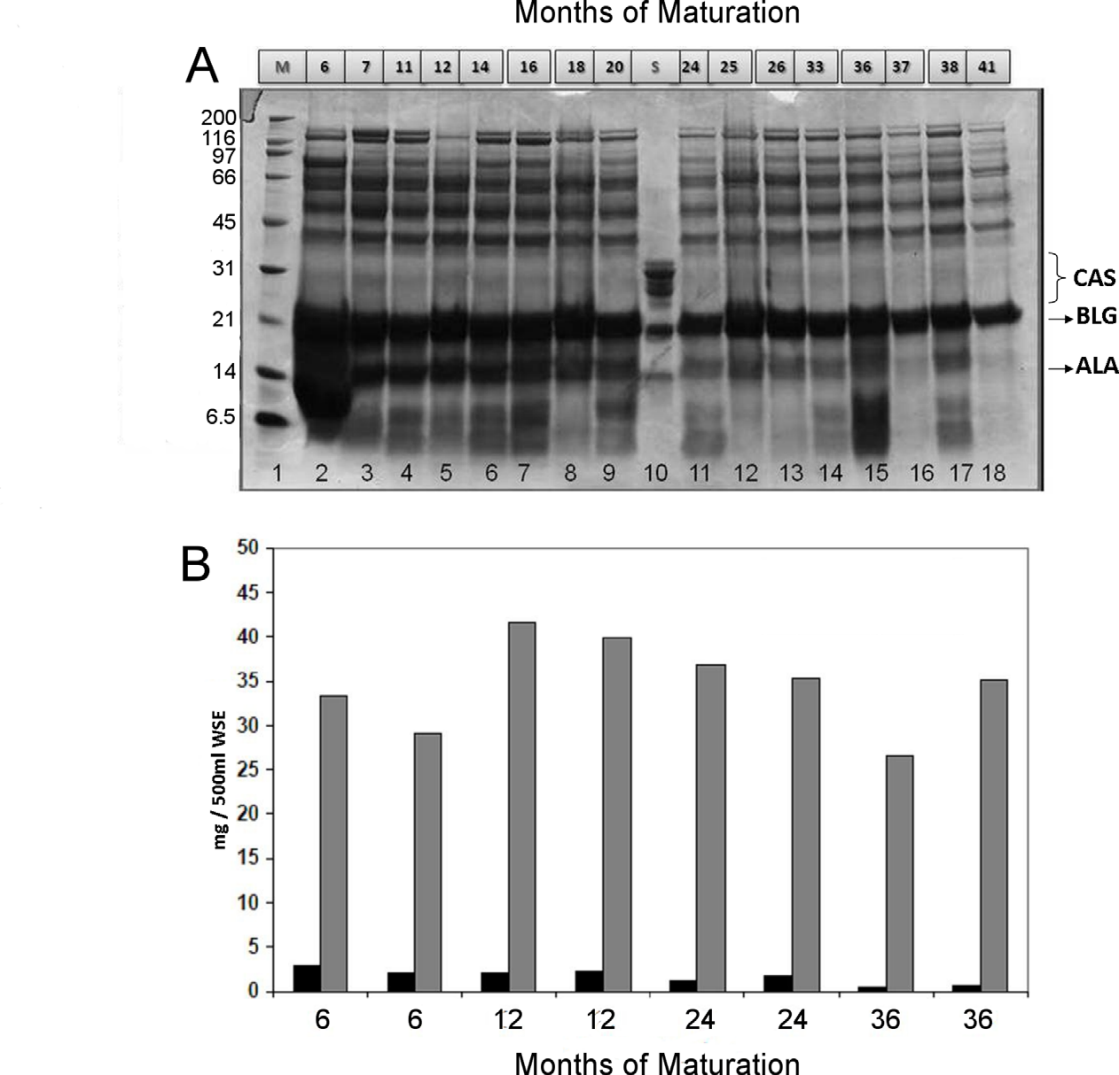
Biochemical evaluation of cow’s milk proteins in *Parmigiano-Reggiano* preparations at different maturation stages. Panel A: SDS-PAGE of *Parmigiano-Reggiano* (PR) water soluble extracts at different maturation time (6–41 months). Extracts from different producers, as detailed in Table 3, are shown to give a comprehensive description of maturation effect on (PR); Lane 2: A-6-1-a; Lane 3: D-7-1-a; Lane 4: F-11-1-b; Lane 5: B-12-1-a; Lane 6: C-14-1-a; Lane 7: A-16-1-a; Lane 8: B-18-1-a; Lane 9: B-20-1-a; Lane11: E-24-1-a; Lane 12: B-25-1-a; Lane 13: D-26-1-a; Lane 14: F-33-1-a; Lane 15: A-36-1-a; Lane 16: E-37-1-a; Lane 17: C-38-1-a; Lane 18: E-41-1-b. The molecular weight marker (M, lane 1) and a standard solution (S, lane 10) containing α-lactalbumin, β-lactoglobulin, α-caseins, β-casein, κ-casein are also included. ALA: α-lactalbumin; BLG: β-lactoglobulin; CAS: casein fraction. Numbers above lanes indicate the month of maturation (lanes 2–9 and 11–18); numbers below indicate lanes. Panel B: Amounts of α-lactalbumin and β-lactoglobulin in the PR aqueous extracts having different maturation ages, as measured by UHPLC/ESI-MS. Some of the analyzed samples from different producers are shown. Preparations shown in the graph can be identified in Table 3 as follows, from left to right: 6 months: A-6-1-a; 7 months: B-6-1-a; 12 months: B-12-1-a; 12 months: D-12-1-a; 24 months: A-24-1-a; 24 months: C-24-1-a; 36 months: A-36-1-a; 36 months: B-36-1-a. Values are expressed as mg/100 gr Water Soluble Extract, and reported as average of two independent analyses (standard deviations aprox 10%). Black bars: α-lactalbumin (ALA); Grey bars: β-lactoglobulin (BLG).

**Figure 2:**
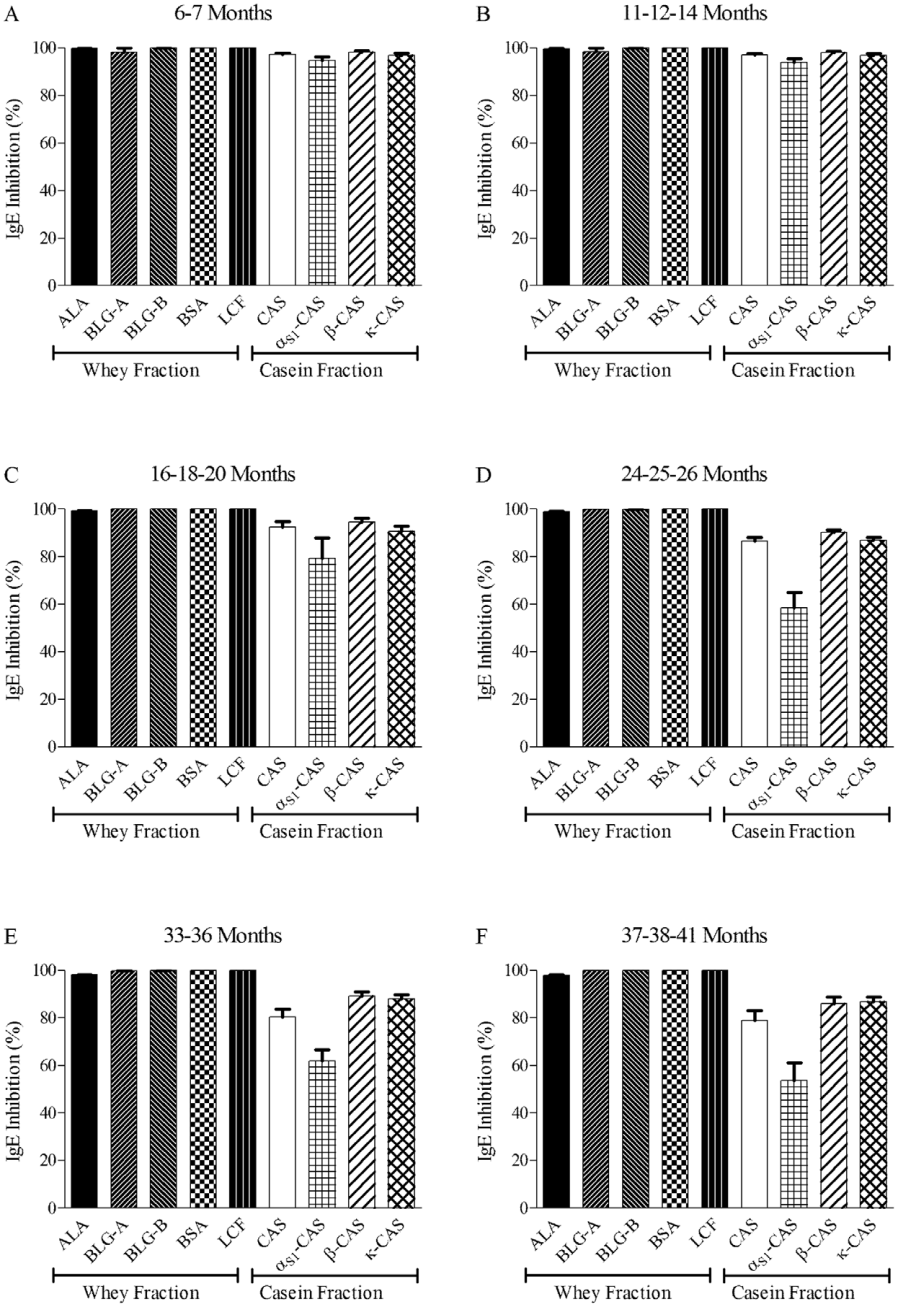
Single Point Highest Inhibition Achievable assay (SPHIAa) results obtained using a pool of sera containing IgE for all milk allergen specificities and *Parmigiano-Reggiano* preparations at different maturation stages. Allergen short names are as in Table 1. Error bars indicating variability among samples from 6 dairies are reported above each allergen result.

### Immunochemical Characterization of PR Water Soluble Extracts

The qualitative allergen composition of each PR WSE was investigated by the Single Point Highest Inhibition Achievable assay (SPHIAa) [17] performed by detecting IgE on the ISAC 89 microarray (VBC-Genomics, Vienna, Austria), where purified natural milk allergens are immobilized. The ISAC 89 microarray was used as, along with other milk allergens, it bears purified CAS, α_S1_-CAS, β-CAS, κ-CAS as well as the two BLG natural variants (Table 1). A pool of sera of allergic subjects known having IgE for all the above allergen specificities was used. Serum donors were not the same patients enrolled in the study because to perform all inhibitions a quite large amount of serum had to be used, thus requiring a higher number of donors. As far as the pooled sera were used as probes for allergen detection in extract by the SPHIAa, sera selection did not influence the experimental outcome. The same pool was used throughout the study for all IgE inhibition experiments. Fifteen µl of the serum pool were incubated overnight with the PR WSE both in the first set of experiments comparing the 92 different PR preparations and in the experiments using digested PR WSE as detailed in Table 3. As non-inhibited control the same serum pool was incubated with D-PBS (Euroclone, Milan, Italy). For the SPHIAa experiments as reported above, using digested PR, preparations from four producers at three maturation stages, young, medium and aged, respectively, were used, as shown in Table 3. ISAC 89 testing for IgE was performed as previously reported [14].

**Figure 3:**
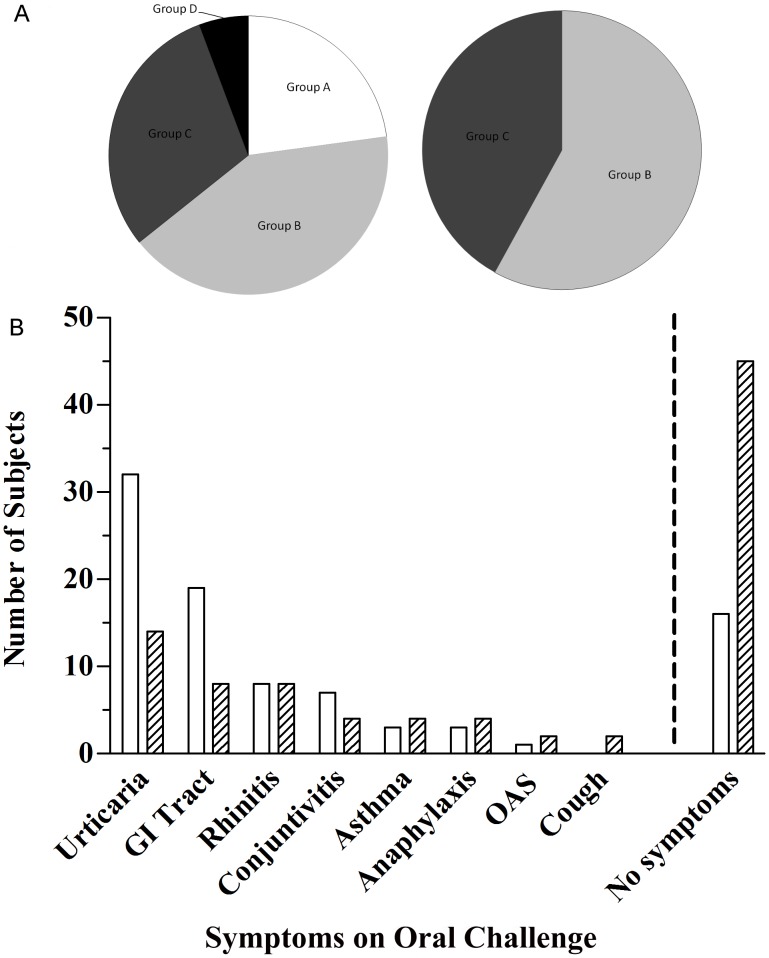
*In vivo* test reactivity distribution and symptoms recorded on DBPCFC with fresh cow’s milk and OFC with 36 months maturated *Parmigiano-Reggiano*. Panel A, left graph, includes all 70 patients either tested or not for both milk and PR preparations. Group A includes Cow’s Milk (CM) and *Parmigiano-Reggiano* (PR) Tolerant subjects: 16 (22.86%); Group B includes CM Reactive PR Tolerant subjects: 29 (41.43%); Group C includes CM and PR Reactive subjects: 21 (30%); Group D includes CM Reactive PR not tested subjects: 4 (5.71%). Panel A, right graph, includes the 50 patients who reacted to CM DBPCFC and differently to PR OFC. Group B includes CM Reactive PR Tolerant subjects: 29 (58%); Group C includes CM and PR Reactive subjects: 21 (42%). Panel B includes the 66 patients tested by both CM DBPCFC and PR OFC; White bars: symptoms recorded after CM DBPCFC in 50 reactive subjects; Hatched bars: symptoms recorded after PR OFC in 21 reactive subjects; The four subjects not tested with PR OFC are not included in the graph. GI Tract: symptoms related to the gastrointestinal tract including vomiting and diarrhea, excluding oral allergy syndrome (OAS). Urticaria, GI tract symptoms and no symptom prevalence differed by the Fisher’s exact test in a statistically significant way for p<0.0017, 0.0297, 0.0001, respectively.

### Statistics

Descriptive statistics included relative frequencies for qualitative and quantitative variables. Specific IgE and SPT results were expressed using median values. Graphpad Prism version 5.02 has been used for advanced statistics and graphs (Graphpad Software Inc., La Jolla, CA, USA). Fisher’s exact test has been used to compare prevalence. To assess test performances, receiver operating characteristic (ROC) analyses were performed using MedCalc (MedCalc SW, Mariakerke, Belgium) on SPT, ImmunoCAP and ISAC specific IgE data, using both CM DBPCFC and PR OFC as gold standard. Sensitivity and specificity, proving the diagnostic performance of each test, were estimated at the optimal cut-off level. Statistically significance level was set at a p value <0.05.

**Table 4:** Demographics and *in vivo* and *in vitro* diagnostic test results in patients referring to our allergy Centre with a previous diagnosis of cow’s milk allergy.

	DBPCFC	OFC	Subjects	Demographics		Total IgE								
Groups	CM	PR	N	Age°	F/M	IU/ml°								
A	Neg	Neg	16	5	6/10	632								
B	Pos	Neg	29	3.9	17/12	1257								
C	Pos	Pos	21	3.6	5/16	424								
D	Pos	N.T.	4	2.3	4/0	393								
	**DBPCFC**	**OFC**	**P-P** *	**Skin Prick Test** *				**IgE ImmunoCAP** *				**IgE ISAC** *		
**Groups**	**CM**	**PR**	**CM**	**CM**	**ALA**	**BLG**	**CAS**	**CM**	**ALA**	**BLG**	**CAS**	**ALA**	**BLG**	**CAS**
A	Neg	Neg	75	56	44	56	31	75	50	38	44	13	6	19
B	Pos	Neg	90	69	54	65	29	93	62	66	76	48	17	41
C	Pos	Pos	95	80	85	100	62	100	86	90	90	67	76	71
D	Pos	N.T.	100	50	100	75	50	100	100	75	75	75	25	25

CM: Cow’s milk; PR: *Parmigiano-Reggiano* cheese;

Group A: CM and PR tolerant; Group B: Allergic to CM, PR tolerant; Group C: Allergic to CM and PR; Group D: Allergic to CM, but not tested for PR OFC.

Allergen short names are as in Table 1. P-P: prick-prick technique with fresh CM.

* Values are expressed as percentage of all test results in the group, considering positive values above each specific test cut-off (P-P and skin prick test >7 mm^2^, IgE ImmunoCAP>0.35 kUa/l, IgE ISAC 0.01 kUa/l).

°Age and total IgE are expressed as medians in years and IU/l, respectively.

**Table 5:** ROC analyses of performed tests using cow’s milk DBPCFC and PR OFC as gold standards.

Cow’s Milk DBPCFC		Criterion§	Sensitivity	Specificity	+LR	−LR	PPV	NPV
	Total IgE	>101	66.0	56.2	1.51	0.60	82.5	34.6
Prick-Prick test	CM*	>56.3	64.0	68.7	2.05	0.52	86.5	37.9
Skin prick test	CM°	>2.8	86.0	43.7	1.53	0.32	82.7	50.0
	ALA	>16	64.0	75.0	2.56	0.48	88.9	40.0
	BLG	>13.5	72.0	62.5	1.92	0.45	85.7	41.7
	CAS	>17.7	32.0	93.7	5.12	0.73	94.1	30.6
ImmunoCAP	CM	>0.52	94.0	50.0	1.88	0.12	85.5	72.7
	ALA	>1.02	58.0	81.2	3.09	0.52	90.6	38.2
	BLG	>0	90.0	50.0	1.80	0.20	84.9	61.5
	CAS	>0.44	82.0	62.5	2.19	0.29	87.2	52.6
ISAC	ALA	>0	56.0	87.5	4.48	0.50	93.3	38.9
	BLG	>0	40.0	93.7	6.40	0.64	95.2	33.3
	CAS	>0	54.0	81.2	2.88	0.57	90.0	36.1
***Parmigiano*** **-** ***Reggiano*** ** OFC**		**Criterion** §	**Sensitivity**	**Specificity**	**+LR**	**−LR**	**PPV**	**NPV**
	Total IgE	≤628	85.7	33.3	1.29	0.43	37.5	83.3
Prick-Prick test	CM*	>59,3	90.5	62.2	2.39	0.15	52.8	93.3
Skin prick test	CM°	>26	76.2	66.7	2.29	0.36	51.6	85.7
	ALA	>16	80.9	57. 8	1.92	0.33	47.2	86.7
	BLG	>28.26	80.9	73.3	3.04	0.26	58.6	89.2
	CAS	>17.7	57.1	88.9	5.14	0.48	70.6	81.6
ImmunoCAP	CM	>3.99	85.7	71.1	2.97	0.20	58.1	91.4
	ALA	>1.93	76.2	80.0	3.81	0.30	64.0	87.8
	BLG	>2.54	66.7	84.4	4.29	0.39	66.7	84.4
	CAS	>5.25	76.2	82.2	4.29	0.29	66.7	88.1
ISAC	ALA	>0.14	66.7	73.3	2.50	0.45	53.8	82.5
	BLG	>0.15	71.4	91.1	8.04	0.31	78.9	87.2
	CAS	>0.43	66.7	75.6	2.73	0.44	56.0	82.9

Allergen short names are as in Table 1.

DBPCFC: Double blind placebo controlled food challenge;

OFC: Open food challenge;

PR: *Parmigiano-Reggiano;*

* Fresh cow’s milk tested by skin prick-prick technique;

° Cow’s milk extract tested by skin prick test;

§ Criteria for applied tests are reported depending on specific test units: Total IgE  =  IU/l; Skin prick test  =  mm^2^; IgE by ImmunoCAP and ISAC  =  kUa/l;

+LR: Positive likelihood ratio; −LR: Negative likelihood ratio; NPV: Negative predictive value;

PPV: Positive predictive value.

## Results

### Casein and Whey Protein Content of PR Water Soluble Extracts

SDS-PAGE of PR WSE samples was obtained. Sixteen samples, received from different producers and having different maturation times, are reported as an example in Figure 1, panel A, to provide an overall picture of the maturation effect on PR. All other samples were consistent with those in the figure (data not shown). The most abundant proteins of the extracts were found to be whey proteins, ALA and BLG, whereas CAS where nearly absent in all the samples (Figure 1, panel A). The most intense protein band, corresponding to the BLG MW, seems to be resistant to PR proteolytic degradation, giving intense bands also in very aged samples, whereas the protein having a MW of about 14 kDa, deemed to be ALA, seems to be partly degraded along the maturation. Quantification of the two whey proteins, performed by UHPLC/ESI-MS on several samples, confirmed what described above from SDS-PAGE results, indicating for intact BLG a content ranging from 30 to 40 mg/500 ml of PR WSE, which was found to be independent from the maturation time, whereas a much lower content of ALA was detected, of about 3–5 mg/500 ml of WSE in the younger samples, which reduced to 1 mg or less in the most aged samples. Figure 1, panel B shows some examples among the 92 analyzed; all other samples were consistent with those in the figure (data not shown).

**Figure 4:**
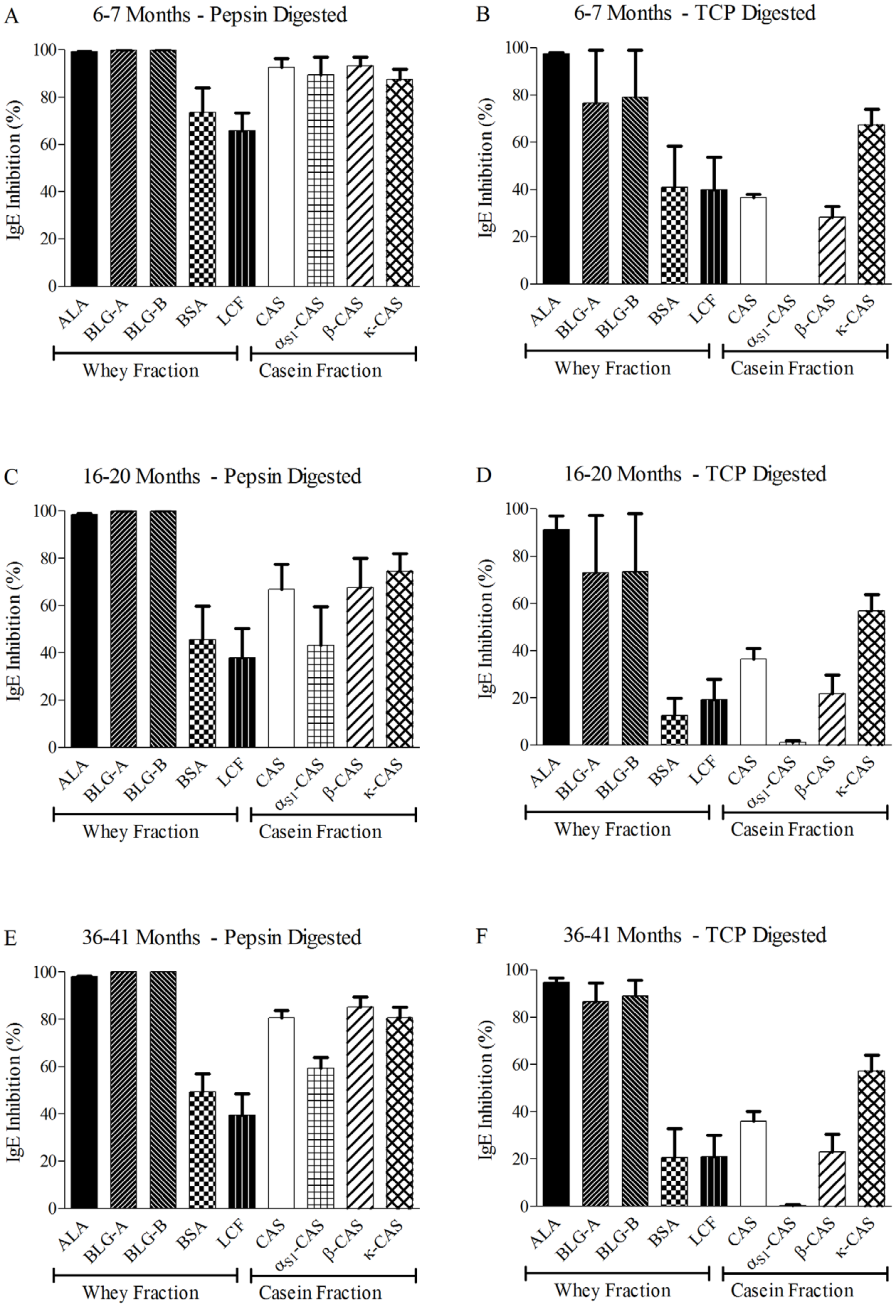
Single Point Highest Inhibition Achievable assay (SPHIAa) IgE experiments with simulated digestions of *Parmigiano-Reggiano* water soluble extracts at three different maturation stages. Results are the average of water soluble extracts obtained from four producers. Error bars, indicating variability among four assays, are reported above each allergen result. TCP: Trypsin, Chimotrypsin, Pepsin.

### Peptide Content of PR Water Soluble Extracts

The WSE were also analyzed by UHPLC-ESI/MS in order to determine the main composition of the peptide fraction. Focusing only on the most abundant peptides, the compounds giving a signal intensity equivalent to at least 30% of the signal of the most abundant compounds were considered. Several peptides were traced and identified in all compounds. Peptides could be divided in two groups: proteolytic compounds, derived from CAS breakdown, where only α_S1_-CAS and β-CAS fragments were identified, being these proteins the most abundant in the CM CAS fraction, and non-proteolytic aminoacyl derivatives, derived from the recombination of the free amino acids in unusual dipeptide like structures (γ-glutamyl-amino acids, lactoyl-amino acids, pyroglutamyl-amino acids) [18].

**Figure 5:**
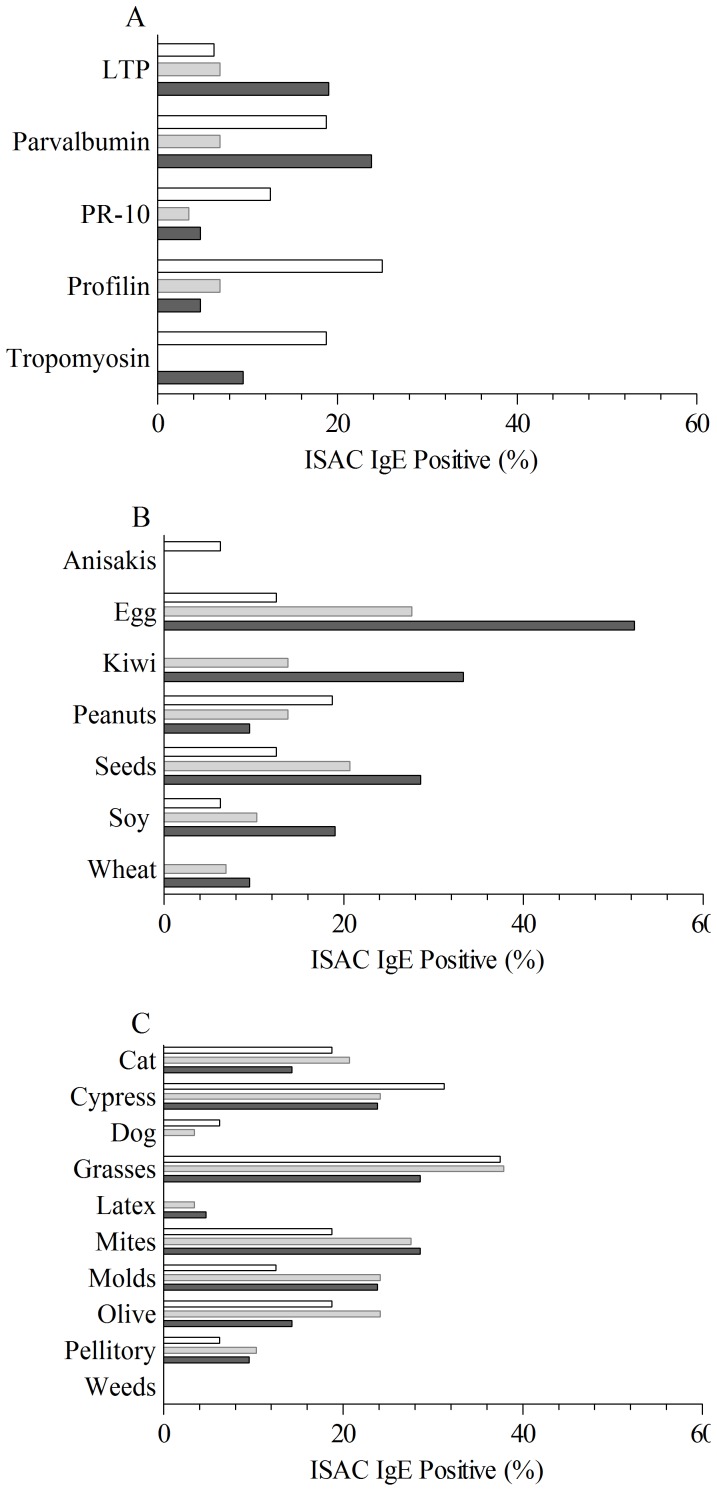
Additional allergen sensitizations detecting IgE using the ISAC103 microarray system in the three CM sensitised subsets depending on their response to CM and PR oral challenges: A (CM- PR-: white bar); B (CM+ PR−: light grey bar); Group C (CM+ PR+: dark grey bar). At least one positive IgE result for one of the following allergens has been evaluated for grouping by allergenic molecule groups and allergenic sources: Panel A - LTP: Cor a 8, Pru p 3; Parvalbumin: Cyp c 1, Gad c 1; PR-10: Act d 11, Api g 1, Ara h 8, Bet v 1, Cor a 1.0101, Cora 1.0401, Dau c 1, Mal d 1, Pru p 1, Gly m 4; Profilin: Bet v 2, Hev b 8, Mer a 1, Ole e 2, Phl p 12; Tropomyosin: Der p 10, Ani s 3, Pen a 1, Pen i 1, Pen m 1; Panel B - Anisakis: Ani s 1; Hen’s egg: Gal d 1, Gal d 2, Gal d 3, Gal d 5; Kiwi: Act d 1, Act d 2, Act d 5; Peanuts: Ara h 1, Ara h 2, Ara h 3; Seeds: Ana o 2, Ber e 1, Cor a 9, Ses i 1; Soy: Gly m 5, Gly m 6; Wheat: Tri a 18, Tri a 19, Tri a Gliadin; Panel C - Cat: Fel d 1, Cypress: Cry j 1, Cup a 1; Dog: Can f 1, Can f 3; Grasses: Phl p 1, Phl p 2, Phl p 4, Phl p 5, Phl p 6, Phl p 11; Latex: Hev b 1, Hev b 3,, Hev b 5, Hev b 6; Mites: Der p 1, Der p 2, Der f 1, Der f 2, Eur m 2; Moulds: Alt a 1, Alt a 6, Asp f 1, Asp f 2, Asp f 3, Asp f 4, Asp f 6; Olive: Ole e 1; Pellitory: Par j 2; Weeds: Art v 1, Art v 3, Amb a 1, Sal k 1. Detailed information about the nature of additional IgE positive allergens detected by ISAC microarray system can be obtained from www.allergome.org.

The quantitative trends observed were quite clear: proteolytic compounds deriving from CAS fragmentation were found to be the most abundant in the less aged samples (6 months) and then continuously decreasing with the increasing of the maturation time, whereas non-proteolytic compounds always increased or maintained a constant amount by increasing the maturation time (data not shown).

These results on the composition of peptide fraction were in perfect agreement with previous works on PR WSE [12], [13] and on recent report detailing the evolution along time of the peptide fraction [Sforza S, Cavatorta V, Lambertini F, Galaverna G, Dossena A et al. J Dairy Sci 2012; accepted for publication].

### Immunochemical Features of PR Water Soluble Extracts

In agreement with the molecular composition reported above, the main results of SPHIAa experiments performed on the 92 PR WSE are shown in Figure 2 and can be summarized as follows: the tested PR WSE samples showed to contain all proteins known as allergens, including milk whey proteins, as all preparation were equally able to exert IgE inhibition on allergens immobilized on the ISAC microarray (Figure 2). No major differences were observed depending on the producer. Extracts of PR matured up to 20 months have an almost intact ability to inhibit specific IgE binding on all ISAC allergens. This result implies that all CM IgE-binding epitopes recognized on ISAC allergens by the serum pool are preserved intact in “young” PR preparations. This applies to all CAS and whey proteins (Figure 2, panel A to C). PR extracts older than 20 months behaved on whey proteins similarly to those of 20 months or younger, whilst for CAS a decreasing IgE inhibition ability was found as a function of PR maturation (Figure 2, panels D to F). This behaviour is more pronounced for α_S1_-CAS, whose IgE binding inhibition gradually decreases to 45% with PR samples of 37–41 months maturation, whilst the IgE binding on β-CAS and κ-CAS slightly decreased reaching an 83–85% inhibition value.

### Oral Challenge with PR

Considering SPT, CM and CM allergen ImmunoCAP IgE and ISAC IgE performed tests, all 70 patients reacted to at least one allergen of at least one of the performed test. As shown in Figure 3, panel A left part, the analysis of the CM DBPCFC and PR OFC allowed us dividing the 70 CM sensitized patients in four groups: group A, 16 patients, tolerated both CM and PR; group B, 29 patients, tolerated PR only; group C, 21 patients, reacted to both CM and PR, while 4 patients in group D, reacted to CM but refused to undergo PR OFC thus dropped off the study. Overall, 29 of 50 patients allergic to CM (58%) tolerated 36 months maturated PR (Figure 3, panel A right part). Symptoms of the patients reacting on DBPCFC to CM and OFC to PR are shown in Figure 3, panel B. Statistical analysis is reported in the Figure 3 legend.

For all performed tests (SPT, ImmunoCAP, ISAC) a common trend was found: percentages of test results above their cut-off gradually increased shifting from group A to B to C (Table 4), with the only exception of CAS SPT. The best marker of PR tolerance seems to be the absence of IgE to BLG on ISAC testing, providing specificity greater than 91.1% at the optimum cut-off of 0.15 kUa/l against PR OFC as gold standard (Table 5).

### IgE Inhibition Using SPHIAa and Digested PR Water Soluble Extracts

Simulated gastrointestinal digestion effects applied to PR WSE at different maturation stages were studied by SPHIAa on IgE. Enzymes without any PR preparation and PR WSE at the same maturation stages without enzyme preparations acted as negative and positive controls, respectively. Recorded results of no IgE inhibition for the former and full IgE binding inhibitions, in an almost overlapping way as in Figure 2, for the latter were obtained (data not shown). SPHIAa for IgE were then carried out on three different maturation ages of PR WSE samples coming from four different dairies, as marked in Table 3. Aggregated data are reported in Figure 4. Whey-derived proteins were differently affected by the two simulated digestions, having no effect at all for ALA, little or even doubtful effect for both BLG variants, and a progressive additive effect on BSA and LCF by both pepsin and TCP digestions, reaching an almost total loss of the IgE binding inhibition capacity for the average and the most aged PR WSE preparations under TCP digestion (Figure 4). Almost the same effect as described for BSA and LCF was recorded for CAS, with a different behaviour of the three purified CAS. α_S1_-CAS, already having its IgE binding inhibition capacity partially reduced by PR maturation (Figure 2), totally lost its IgE inhibitory capability in a progressive and increasing manner with increasing PR maturation age, mainly under TCP digestion (Figure 4). A very partial effect was also recorded during pepsin digestion. ß-CAS and κ-CAS, whose IgE binding inhibition capability was almost not affected by PR maturation (Figure 2), were not influenced by the pepsin digestion, whereas had their IgE inhibition activity reduced by TCP digestion, more pronounced though not total for ß-CAS (Figure 4).

### PR Medium-long Term “Real Life” Tolerance

All PR tolerant subjects were in the follow up phase. Parents were instructed to continue using the 36 months maturated PR at home by using the commercially available preparations as certified by the producer on the marketed packages. No specific weekly schedule of PR intake was given assuming its use as normal in Italians daily life. Overall follow up period lasted from December 2009 up to December 2011, with the shortest observation time of six months just for one child. None of the PR tolerant subjects reported any minor or major complaint in the follow up phase, all using PR as in the usual family habit.

### IgE Detection Toward Additional Non-CM Food and Inhalant Allergens

Testing 103 allergenic molecules for IgE using the microarray approach allowed us to detect sensitizations also to other important allergens from other sources as shown in Figure 5. Allergenic molecules were considered either as markers of allergen group sensitization (Figure 5, Panel A) or as markers of sensitization to a given allergenic source (Figure 5, Panel B–C; *i.e.* Gal d 1, 2, 3, and 5 for hen’s egg allergy, Phl p 1, 2, 4, 5, 6, and 11 for grass pollen allergy). Additional details on allergens are given in Figure 5 legend. Eleven patients had no additional IgE detectable sensitizations on the ISAC103; 5 were in the group A, 5 in the group B, and 1 in the group C. Evaluating the 55 subjects being positive for at least one non-CM allergen, a general trend was recorded for having higher sensitization prevalence, mainly in group C, to some of the other food allergens, namely the markers for hen’s egg, seeds excluding peanuts, soy, and kiwifruit, or to food panallergens like lipid transfer proteins from plant-derived foods and parvalbumins from fishes. Such trend was not recorded for allergens causing sensitization by inhalation like those from pollen, mites, mammal epithelia, and moulds.

## Discussion

Although other authors already reported tolerance to baked CM proteins in CM allergic children [7], [19], [20], we herein prove for the first time how CM allergic children can tolerate a dairy product as PR cheese, lacking CM high temperature processing. Although all CM allergenic proteins extracted from PR in its various maturation stages largely retained their ability to bind IgE, the clinical findings prompted us to investigate the clinical tolerance mechanism of this non-baked product. Among the CM proteins, CAS seems to behave differently from the others, as the proteolytic enzymes activity during the maturation process reduces, at least partially, their IgE binding capability. The presence of CAS derived peptides indicate that an intense proteolytic process on the CAS fraction is continuously acting, degrading the full proteins and the peptides derived from them, making aged PR very poor in CAS in their native structure. The absence of CAS in the extracts might thus be due in part to the poor CAS solubility in water, but mostly to their proteolytic degradation which takes place during PR manufacturing and maturation; actually, older samples were accordingly poorer in CAS than younger ones.

Conversely, no peptides derived from whey proteins were observed, indicating that the proteolysis does not affect these proteins, and actually whey protein content seems not to be affected by the maturation time. As for CAS, whey proteins retain an almost intact ability to bind IgE also in very aged PR preparations.

Whey proteins were unexpectedly present in all WSE, albeit the fact that during cheese making only CAS precipitate in the curd and become part of the cheese, whereas most of the whey proteins should remain in solution and used for other diary production purposes. Anyway, since some whey is always included in the curd, it is quite obvious to presume that part of the whey proteins become trapped in the PR.

As shown in the first part of simulated digestion, pepsin treatment of WSE further reduces the IgE binding inhibition to CAS, and start decreasing the one to BSA, and to LCF. The effect of pepsin was better seen when it acted on aged PR WSE. Digestion with TCP, however, can greatly reduce the IgE binding to CAS, almost abolishing the one to α_S1_-CAS, markedly reducing the one to β-CAS, BSA, LCF, and reducing by a 50% the one to κ-CAS. Overall, PR maturation combined with gastro-duodenal digestion brought to a marked decrease of the IgE binding inhibition capability of WSE. Opposite to CAS and other whey proteins, ALA and BLG IgE binding inhibition were almost not affected by the combined PR and simulated digestions. ALA, BLG and CAS resistance/sensitivity to digestion has been already reported by many authors in the past and recently [2], [21], [22], and it seems to largely depend on the kind of enzymatic treatment used and additional experimental conditions (*e.g.* heating) [2], [23], [24]. Reduction in the antigenicity of CM proteins can be also achieved using fermentation with lactic acid bacteria [25], the same microorganisms added to produce PR, though in experimental models BLG seems to be fully degraded [26], [27]. The clinical outcome of our study clearly shows the beneficial effect of the PR production procedures, though the clinical tolerance seems to be achieved combining the partial proteolysis degrading some CM proteins in highly aged PR, the gut digestion and a specific IgE reactivity to allergenic molecule defined by diagnostic profiling with the ISAC microarray. BLG IgE reactivity seems to play a critical role at this regard as shown by their presence in sera of PR-reactive patients. These findings are in line with those previously reported of preserved IgE binding by BLG peptides [28].

IgE detection by SPHIAa is a simple method to identify allergenic molecules in foods [17]. It provides a preliminary estimate of allergen presence in the source, though the result largely depends on concentration, affinity, and avidity of the allergenic molecules under study. Inhibition can be achieved by a molecular structure with preserved IgE epitopes as shown for BLG and CAS by Cerecedo et al. [29], not necessarily still biologically active. Hence, as in the present study, SPHIAa results suggest the presence of peptides and other structures still able to bind CM allergic patients’ IgE. These IgE binding structures might not trigger mast cell activation. Nevertheless, the usefulness of a multiplexed IgE inhibition retains its validity as it gives the full profile of proteins present or not in a preparation. Notably, in our experiments the availability of fractionated CAS on ISAC 89 lead to differentiate the sensitivity to digestion of each of them. As reported by Bernard et al. [30], IgE reactivity to different CAS is part of the personal profile of each CM allergic patient, and CAS should be not considered as unique entity. The biochemical and immunochemical experiments run in the present study show an unexpected presence of seemingly intact whey proteins in matured PR. Whey, a by-product from cheese or CAS manufacture, contains less than 1% proteins: ALA, BLG, immunoglobulins and protease peptones, as well as several minor proteins including lactoferrin, glycoproteins, lactoperoxidase and transferrin [1]. Recognition of still present whey proteins could thus explain PR allergy. Although CM allergy is often due to more than one epitope present on different CM proteins, up to now no specific CM protein has ever been identified as ‘major and unique milk allergen’ [31], [32]. In terms of PR tolerance, a good marker has been identified in BLG negative IgE results obtained by ISAC, with specificity greater than 91% on ISAC test. At the moment we are not aware of the reasons behind a better performance of IgE detection made by ISAC rather than by ImmunoCAP, though we might speculate that a different antigen/antibody interaction kinetic could exist in the micro *versus* the macro system. Even in those favourable cases, where a large rate of success is expected when giving patients with fully matured PR, oral food challenge in a supervised setting to assess PR tolerability in CM allergic children is strongly suggested. PR allergic reaction, though rarer, could be very severe, as shown in the present study, being PR a CM protein concentrates. Conversely, specific markers of PR clinical reactivity are still to be identified.

The tested PR tolerant children are going ahead consuming PR without any problem. The clinical follow up study is thus supporting that unless the PR product is not a truly industrial one, it achieves “reproducibility” in production as documented by our biochemical and immunochemical evaluations. Further studies are required to verify if regular PR ingestion might anticipate CM tolerance as reported in studies using baked CM products [7]. Published studies on children outgrowing CM allergy have shown levels of CM specific IgE antibodies lower than those of persistent allergic patients [9], [33], therefore a low IgE response to all CM proteins on microarray testing, as reported in the present study, could be a good marker of PR tolerability toward the following CM reintroduction.

As for our previous study on hen’s egg allergic children [10], some differences in the overall allergenic molecule-based diagnostic profile were found when comparing the three groups of children. Beside the problematic CM allergy and not being tolerant to PR ingestion, group C had also a higher number of additional food sensitization, which, with a timely diagnosis, could prevent major allergic reactions to food sources other than CM.

In conclusion, even if SPHIAa results on PR analysis show the presence of either proteins, or peptides or other compounds at different molecular weight still able to bind IgE, a significant proportion (58%) of patients reacting to CM tolerate PR. A possible explanation is the CAS partial hydrolysis induced by the 36 months PR maturation process, additionally digested in the gastro-intestinal tract. Although the best marker of PR tolerance seems to be an IgE BLG negative result on ISAC testing, a fully reliable model to identify patients tolerating PR without a risky oral challenge is still to be finalized. Our combined biochemical, immunological, and diagnostic methods should be encouraged in study protocols exploring CM protein clinical tolerance to allow comparability among different approaches. Reintroduction of a tasty food as PR was highly appreciated by tolerant children and their relatives leading to an improvement of the overall family quality of life. Hence, PR could be added to baked products for CM allergic children management.
